# Tumour-targeted delivery of TRAIL using *Salmonella typhimurium* enhances breast cancer survival in mice

**DOI:** 10.1038/sj.bjc.6605403

**Published:** 2009-10-27

**Authors:** S Ganai, R B Arenas, N S Forbes

**Affiliations:** 1 Program in Molecular and Cellular Biology, University of Massachusetts Amherst, Amherst, MA 01003, USA; 2 Department of Surgery, Baystate Medical Center/Tufts University School of Medicine, Springfield, MA 01199, USA; 3 Pioneer Valley Life Sciences Institute, Springfield, MA 01107, USA; 4 Department of Chemical Engineering, University of Massachusetts Amherst, Amherst, MA 01003, USA

**Keywords:** targeted cancer therapy, spatiotemporal controlled delivery, attenuated *Salmonella typhimurium*, TRAIL, *RecA*

## Abstract

**Background:**

An effective cancer therapeutic must selectively target tumours with minimal systemic toxicity. Expression of a cytotoxic protein using *Salmonella typhimurium* would enable spatial and temporal control of delivery because these bacteria preferentially target tumours over normal tissue.

**Methods:**

We engineered non-pathogenic *S. typhimurium* to secrete murine TNF-related apoptosis-inducing ligand (TRAIL) under the control of the prokaryotic radiation-inducible *RecA* promoter. The response of the *RecA* promoter to radiation was measured using fluorometry and immunoblotting. TRAIL toxicity was determined using flow cytometry and by measuring caspase-3 activation. A syngeneic murine tumour model was used to determine bacterial accumulation and the response to expressed TRAIL.

**Results:**

After irradiation, engineered *S. typhimurium* secreted TRAIL, which caused caspase-3-mediated apoptosis and death in 4T1 mammary carcinoma cells in culture. Systemic injection of *Salmonella* and induction of TRAIL expression using 2 Gy *γ*-irradiation caused a significant delay in mammary tumour growth and reduced the risk of death by 76% when compared with irradiated controls. Repeated dosing with TRAIL-bearing *Salmonella* in conjunction with radiation improved the 30-day survival from 0 to 100%.

**Conclusion:**

These results show the pre-clinical utility of *S. typhimurium* as a TRAIL expression vector that effectively reduces tumour growth and extends host survival.

Selective targeting of tumours enhances therapeutic delivery by increasing the drug concentration in cancer cells while limiting damage to normal tissue (Jain and Forbes, 2001; Forbes, 2006). Many genera of bacteria have been shown to selectively target tumours (Low *et al*, 1999; Forbes *et al*, 2003) and have great promise as adjunctive cancer therapies (Dang *et al*, 2001; Zhao *et al*, 2007). Despite this potential, once these bacterial species have been rendered non-toxic to humans, their toxicity is also limited against cancer cells, lowering their clinical significance (Toso *et al*, 2002; Nemunaitis *et al*, 2003). The utility of these bacterial therapies can be enhanced by engineering controllable expression of a cytotoxic peptide. Because these bacteria preferentially accumulate in tumour microenvironments (Zhao *et al*, 2005; Kasinskas and Forbes, 2006), they can also be manipulated to control the location and timing of cytotoxic peptide expression and further reduce host toxicity.

Four main genera of bacteria have been analysed as anticancer agents (Wei *et al*, 2008) and have been shown to selectively target tumours after intravenous injection: *Salmonella* (Low *et al*, 1999), *Escherichia* (Yu *et al*, 2004; Stritzker *et al*, 2007), *Clostridium* (Lemmon *et al*, 1997; Dang *et al*, 2001; Theys *et al*, 2006), and *Bifidobacterium* (Yazawa *et al*, 2001). To improve their efficacy, these bacteria have been engineered to express numerous proteins, including cytosine deaminase (Theys *et al*, 2001; Dresselaers *et al*, 2003; Dubois *et al*, 2007), endostatin (Lee *et al*, 2004), thrombospondin-1 (Lee *et al*, 2005), TNF-*α* (Nuyts *et al*, 2001b), interleukin-2 (Barbe *et al*, 2005), and antibodies against HIF-1-*α* (Groot *et al*, 2007). Among these, only TNF-*α* is directly toxic to cancer cells. The facultative anaerobes, *Salmonella* and *Escherichia*, have an advantage because they can be administered systemically in their active form, which permits them to penetrate and chemotax through tumour tissue to target specific microenvironments (Zhao *et al*, 2005; Kasinskas and Forbes, 2006). *Salmonella* has particular promise as a cancer therapeutic because it can be manipulated to target quiescent, diffusion-limited regions in solid tumours (Kasinskas and Forbes, 2007) that are p53 deficient (Yu *et al*, 2002) and resistant to chemotherapeutics and radiation therapies (Minchinton and Tannock, 2006; Tredan *et al*, 2007). In addition, *Salmonella* has been shown to preferentially accumulate in tumours over 1000 times more than other organs after systemic injection, a characteristic that would reduce off-target toxicity from bacterially expressed molecules (Low *et al*, 1999; Forbes *et al*, 2003).

Temporal control of gene expression has been previously shown in *Clostridium* (Nuyts *et al*, 2001a, 2001b) and viral vectors (Senzer *et al*, 2004; Mezhir *et al*, 2006). Both of these systems use radiation to trigger gene expression because of its ability to penetrate through tissue and trigger promoters that respond to DNA damage (Anderson and Kowalczykowski, 1998). In *Clostridium,* the prokaryotic promoter to *RecA* has been shown to control TNF-*α* production after irradiation (Nuyts *et al*, 2001a, 2001b). Similarly, temporal control of TNF-*α* expression has been shown using an adenoviral vector that encodes the eukaryotic *Egr1* promoter upstream of TNF-*α* (Mezhir *et al*, 2006). In phase I clinical trials, this system was well tolerated (Senzer *et al*, 2004) and has shown to regress tumours (McLoughlin *et al*, 2005); however, it requires intratumoural injection to ensure its local effect, and is thus limited by access.

TNF-related apoptosis-inducing ligand (TRAIL) is a promising cancer therapeutic because it has been shown to selectively induce apoptosis in cancer cells when compared with normal tissue (Yagita *et al*, 2004). TRAIL is effective in many cancer cells, including colon, breast, lung, prostate, renal, ovarian, bladder, glioma, and pancreatic tumours (Walczak *et al*, 1999; Hylander *et al*, 2005; Shanker *et al*, 2008). Similar to TNF-*α*, TRAIL exerts an effect by the death receptor pathway of apoptosis, which activates caspase-8 and leads to activation of caspase-3, an important apoptotic mediator (LeBlanc and Ashkenazi, 2003). As an expressible peptide, TRAIL has great potential because it is non-toxic to most normal tissues, unlike TNF-*α* (Ashkenazi *et al*, 1999; Cretney *et al*, 2006), and because bacterially produced TRAIL has been shown to be cytotoxic to cancer cells (Kim *et al*, 2004). However, TRAIL has a limited role as a blood-borne therapy because of its rapid renal clearance and short half-life (Kelley *et al*, 2001). Despite its specificity towards tumours over most tissues, TRAIL may also induce hepatic cell death (Zheng *et al*, 2004), which suggests that an approach that allows for its selective delivery into tumours would avoid potential hepatotoxicity.

We have designed a targeted cancer therapy that provides spatiotemporal control of cytotoxic protein delivery by using *Salmonella typhimurium* as a vector to deliver TRAIL into tumours. To create a radiation-inducible system, the TRAIL gene was coupled to the promoter sequence for *RecA*, a gene involved in the prokaryotic SOS response to DNA damage (Anderson and Kowalczykowski, 1998). *RecA* promoter stimulation was shown using fluorometry, and TRAIL expression, secretion, and function were shown using immunoblotting and mammalian cell culture. A syngeneic murine breast cancer model was used to show the ability of radiation-inducible TRAIL secretion using *S. typhimurium* to suppress tumour growth and enhance host survival. Because of the ability to control the spatial and temporal delivery, TRAIL delivery using *S. typhimurium* has a great potential to be an adjunctive treatment strategy for solid tumours.

## Materials and methods

### Development of plasmid constructs

**Figure 1:**
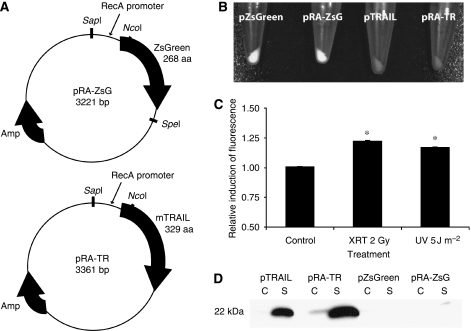
Radiation-inducible prokaryotic expression constructs for ZsGreen and TRAIL. (**A**) The plasmid construct, pRA-ZsG, has the *S. typhimurium* promoter for *RecA* upstream of the green fluorescent protein, ZsGreen. The plasmid construct, pRA-TR, substitutes ZsGreen with the apoptosis-inducing peptide, mTRAIL. (**B**) Fluorescence imaging shows green fluorescence from bacteria electroporated with pZsGreen and pRA-ZsG plasmid constructs. (**C**) Fluorometry showing relative induction by the *RecA* promoter. *S. typhimurium* VNP20009 electroporated with pZsGreen and pRA-ZsG were induced with 2 Gy *γ*-irradiation or 5 J m^−2^ UV irradiation, and then grown at 37 °C, 250 r.p.m. for 4 h. Data were normalised to the bacterial absorbance at 600 nm, and are reported as relative fluorescence of VNP pRA-ZsG compared with VNP pZsGreen. Gene expression is significantly increased with genotoxic damage in comparison with the un-induced control (^*^P<0.05). (**D**) Immunoblot showing TRAIL secretion. 15% SDS–PAGE was performed on 20 *μ*g protein obtained from cytosolic (c) and supernatant (s) fractions of overnight bacterial culture transformed with plasmid constructs. After transfer to PVDF, an immunoblot was performed using 1 : 200 Rabbit anti-TRAIL polyclonal antibody.

A series of prokaryotic-expression plasmids were created that contained either murine TRAIL or the green fluorescent protein, ZsGreen, under the control of the endogenous *S. typhimurium RecA* promoter (Figure 1). Plasmid cloning was performed using *Escherichia coli DH5-α* (Invitrogen, Carlsbad, CA, USA) and all restriction endonucleases were obtained from New England BioLabs (Ipswich, MA, USA). To create a prokaryotic expression construct specific for *S. typhimurium*, the *RecA* promoter sequence (−91 to +84) was PCR amplified from the *S. typhimurium* genome (McClelland *et al*, 2001) using the forward primer 5′-CACCAGCGGAAGAGCGACAGGCGACGACATA-3′ (*Sap*I restriction site underlined) and the reverse primer 5′-AGAACCCATGGACGCTTTCAGTTTGTTTTC-3′ (*Nco*I restriction site underlined). The radiation-inducible, control plasmid, pRA-ZsG (Figure 1A) was created from the pUC backbone of the prokaryotic expression plasmid pZsGreen (Clontech, Mountain View, CA, USA) by subcloning the *RecA* promoter into the *Sap*I and *Nco*I sites to substitute the *lac* promoter. Two plasmids for expression of murine TRAIL were developed, pTRAIL and pRA-TR (Figure 1A), by excising the murine TRAIL fragment from pORF5-mTRAIL (InvivoGen, San Diego, CA, USA) using *Nco*I and *Nhe*I, and subcloning into the *Nco*I and *Spe*I sites on pRA-ZsG.

The construct pQCXIN_mCh was developed by subcloning the *Bam*HI*/Eco*RI digested fragment from pRSET-B_mCherry into the retroviral vector, pQCXIN (Clontech). Appropriate frame and sequence of inserts was confirmed using an ABI Prism 3730xl DNA Sequencer (Genewiz, South Plainfield, NJ, USA).

### *S. typhimurium* transformation using electroporation

The *msbB*^*−*^*purI*^*−*^*xyl*^*−*^*Salmonella typhimurium* strain, VNP20009 (Vion Pharmaceuticals, New Haven, CT, USA), was grown in Luria-Bertani (LB) media at 37 °C until mid-log phase, and then harvested at 4 °C. Cells were made electrocompetent after serial washes in ice-cold 10% glycerol, followed by resuspension in GYT medium (10% glycerol, 0.125% yeast extract, 0.25% tryptone) at 2.5 × 10^10^ cells ml^−1^. Electroporation was performed in 0.2 cm cuvettes after mixing 40 *μ*l competent cells with 25 ng plasmid DNA using a Bio-Rad Micropulser with settings of 2.5 kV, 25 *μ*F, and 200Ω. Bacteria were selected and maintained in LB media with 50 *μ*g ml^−1^ ampicillin.

### Confirmation of *RecA* promoter function using imaging and fluorometry

Bacteria electroporated with ZsGreen plasmid constructs were grown until an OD600 of 0.5, and then exposed to 1.0 mM IPTG, 2 Gy *γ*-irradiation, or 5 J/m^2^ UV irradiation. Bacteria were incubated at 37 °C, 250 r.p.m. for 4 h. Fluorescence was observed using a Maestro *In-Vivo* Imaging System (Cambridge Research & Instrumentation, Inc., Woburn, MA, USA). Fluorescence intensity was quantified using a microplate reader with 485 nm excitation and 535 nm emission filters.

### Measurement of TRAIL expression using immunoblotting

Bacteria electroporated with plasmid constructs were grown overnight in modified M9 Media (0.4% glucose, 1% tryptone, 200 *μ*M purine base, and 50 *μ*g ml^−1^ ampicillin). Bacteria were centrifuged at 15 000 **g**, with aspiration of the supernatant fraction. Secreted proteins from media supernatants were precipitated with 0.1% sodium deoxycholate and 7% trichloroacetic acid, followed by washes in acetone and resuspension in phosphate-buffered saline (PBS). The cell pellet was incubated in bacterial lysis buffer (50 mM potassium phosphate, 400 mM NaCl, 100 mM KCl, 10% glycerol, 0.5% Triton X-100, and 10 mM imidazole, pH 7.8) at 4 °C to extract cytosolic proteins. Protein concentration was determined using a BCA assay kit (Pierce, Rockford, IL, USA). With 20 *μ*g of protein per well, 15% SDS–PAGE was performed, followed by transfer onto PVDF membrane. Membranes were blocked in 5% milk in Tris-buffered saline with 0.1% Tween-20 (TBST), and then incubated overnight at 4 °C in 1 : 200 Rabbit anti-TRAIL polyclonal antibody (Abcam, Cambridge, MA, USA). After serial washes in TBST, membranes were incubated in 1 : 5000 HRP-conjugated goat anti-rabbit polyclonal antibody, and then washed again and visualised using Supersignal chemiluminescent substrate (Pierce).

### Mammary tumour cell lines

Mammary carcinoma 4T1 cells (American Tissue Type Collection, Manassas, VA, USA) were maintained at 37 °C, 5% CO_2_ in RPMI-1640 with 10% fetal bovine serum. Using tumours composed of syngeneic 4T1 cells in BALB/c mice was one of the few appropriate cancer models. It was necessary to use immunocompetent mice because attenuated bacteria have deleterious effects on immunocompromised mice (Forbes *et al*, 2003). Because of rejection by the immune system, only syngeneic tumours will form in immunocompetent mice.

An attenuated mammary carcinoma cell line, 4T1/red, was developed because implanted 4T1 cells invaded the peritoneum and caused diffuse metastatic disease within 3 to 4 weeks. The new 4T1/red cell line was developed by G418 selection after retroviral infection of 4T1 with pQCXIN_mCh viral supernatants, obtained after transformation of the EcoPack 2–293 cell line (Clontech). Tumours formed from 4T1/red cells had an increased doubling time and no longer created pulmonary or hepatic metastases, with decreased intraperitoneal invasion. Using 4T1/red cells allowed for observation of growth of the primary tumour without mice succumbing to bowel obstruction or diffuse metastatic disease. Prevention of metastatic disease enabled a more accurate test of the hypothesis that bacterial treatments influence growth of the primary tumours. Immediately before *in vivo* use, trypsinised 4T1/red cells were resuspended in PBS at 5 × 10^6^ cells ml^−1^.

### Toxicity of bacterial secreted protein on mammalian cell culture

Filtered supernatants were obtained from bacterial strains (VNP pRA-ZsG and VNP pRA-TR) grown in modified M9 Medium (0.4% glucose, 1% tryptone, 200 *μ*M purine base, and 50 *μ*g ml^−1^ ampicillin) and resuspended in 4T1 media at a concentration of 50 ng ml^−1^. The control treatment used sterile modified M9 Medium. Recombinant mouse TNF-*α* (Sigma, St Louis, MO, USA) was also suspended in 4T1 media at a concentration of 50 ng ml^−1^.

### Caspase activity assays

ApoAlert caspase activity assays (Clontech) were conducted on 4T1 cells after application of experimental supernatants or recombinant mouse TNF-*α* (Sigma) at 50 ng ml^−1^ for 24 h. Cell lysates were obtained from 2 × 10^6^ cells and incubated in caspase-3 substrate (DEVD-pNA) or caspase-8 substrate (IEDT-pNA) according to the manufacturer's directions. Validation of the assays was performed by incubating cell lysates from the TNF-*α* treatment group with DEVD-fmk, a caspase-3 inhibitor.

### Annexin V-FITC/propidium iodide flow cytometry

4T1 cells were exposed to experimental treatments for 48 h. Cells were prepared using an Annexin V-FITC apoptosis detection kit II (BD Biosciences, San Jose, CA, USA), according to the manufacturer's directions. Flow cytometry was conducted on 10 000 cells per treatment using a FACSCalibur flow cytometer (BD Biosciences). Normalisation and compensation were performed on unstained controls.

### Syngeneic murine tumour model

Animal care was conducted in accordance with the National Institute of Health guidelines for care and use of laboratory animals. Previous approval from the institutional animal care and use committee of the Baystate Medical Center was obtained. At 8 weeks of age, Balb/c mice received a subcutaneous injection of 50 000 4T1/red cells using a 10 *μ*l Hamilton syringe at the level of the right third mammary fat pad.

### Biodistribution

To determine bacterial biodistribution, 4T1 tumours were grown for 21 days. At this time, mice received systemic injection through tail vein with 100 000 cfu g^−1^ VNP20009. Mice were killed after 48 h. The tumour and liver samples were weighed and then minced in a known volume of PBS until homogenous, followed by plating of serial dilutions on LB agar. After 24- to 48-h incubation at 37 °C, colony-forming units (cfu) were counted to determine bacterial concentration (cfu g^−1^).

### *Salmonella* immunohistochemistry

Tumours were fixed in 10% neutral buffered formalin and embedded in paraffin. Antigen retrieval was performed on rehydrated equatorial tissue sections using an EZ Retriever System with CitraPlus Antigen Retrieval Solution (BioGenex, San Ramon, CA, USA). A DakoCytomation Autostainer (Carpinteria, CA, USA) was programmed according to the manufacturer's settings using an Envision G/2 AP System. Sections were incubated in a 1 : 200 dilution of Rabbit Polyclonal Antibody to Salmonella (Abcam) in 0.2% BSA for 30 min. An alkaline phosphatase polymer was applied for 30 min, followed by staining with Permanent Red. Sections were counterstained with hematoxylin. Positive controls were performed on a tumour with intratumoural injection of VNP20009.

### Image acquisition

An Olympus IX71 Inverted Epi-fluorescence Microscope (Center Valley, PA, USA) equipped with a Ludl Motorised Z-Stage (Hawthorne, NY, USA), a monochromatic Hamamatsu cooled-CCD Digital Camera (Hamamatsu City, Japan), and a CRI MicroColor trichromatic filter (Woburn, MA, USA) was used to acquire images at × 10 objective lens magnification from immunohistochemically-labeled slides. A script in IPLab (version 3.71, BD Biosciences) was used to automate image acquisition and assemble a tiled montage of individual images comprising three RGB channels, creating macroscopic composite colour images of entire tumour sections.

### Tumour response to bacterial treatment

After 21 days of tumour growth, Balb/c mice received systemic injection through tail vein with 100 000 cfu g^−1^ VNP pRA-ZsG, VNP pRA-TR, or PBS (control). After 2 days, subgroups of mice received a single dose of 2 Gy whole-body *γ*-irradiation by exposure to a Gammator-50 ^137^Cs Source (Radiation Machinery Co., Parsippany, NJ, USA). This time point was determined from observations in 4T1 tumours showing that bacteria were furthest away from perfused vasculature at 48 h. Tumour volume was determined every 2 days with measurements obtained from an electronic vernier caliper using the equation (length)(width)(height)*π*/6. Mice with tumours <75 mm^3^ at 21 days of tumour growth were excluded from analysis. Time of killing was determined when mice were moribund or tumour volume exceeded 1000 mm^3^. Follow-up was limited to 30 days.

### Statistical analysis

Data are reported as means with their 95% confidence intervals in parentheses. Hypothesis testing was performed using Student's *t*-test with significance determined by *P*<0.05. Regression analysis was performed according to logarithmic functions for tumour growth. Survival analysis was performed using Kaplan–Meier curves, with comparisons between groups made using the log-rank test. Hazard ratios were determined using Cox's proportional hazards analysis.

## Results

### TRAIL expressed using *S. typhimurium* induces apoptosis and cell death

Gene expression from the created plasmids was confirmed using fluorescence imaging and immunoblotting (Figures 1B–D). Figure 1B shows the presence of green fluorescence from VNP pZsGreen and VNP pRA-ZsG. To assess the ability of the *RecA* promoter to be triggered by genotoxic damage, the fluorescence of VNP pRA-ZsG was compared with VNP pZsGreen. The ratio of fluorescence induction by the *RecA* promoter to the *lac* promoter significantly increased by 22% at 4 h after 2 Gy *γ*-irradiation in comparison with non-induced controls (*P*<0.05; Figure 1C). Fluorescence present in the non-irradiated control is because of the low-level constitutive expression of the *RecA* promoter (Nuyts *et al*, 2001a). In bacterial cultures, immunoblots for TRAIL protein showed that engineered *S. typhimurium* produced TRAIL that was predominantly secreted into the media supernatant and only minimally retained in the bacterial cytoplasm (Figure 1D). Although not quantitative, this immunoblot shows that pTRAIL and pRA-TR produce considerable amounts of secreted mTRAIL.

**Figure 2:**
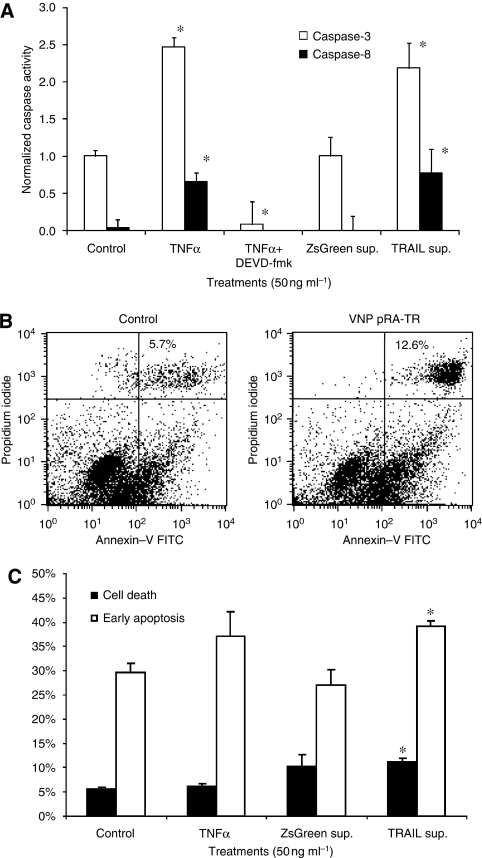
*In vitro* effects of secreted proteins from transformed *S. typhimurium* on 4T1 mammary carcinoma cells. Filtered supernatants were acquired from VNP pRA-ZsG (ZsGreen sup.) and VNP pRA-TR (TRAIL sup.) after growth for 4 h in minimal medium after induction with 5 J m^−2^ UV irradiation. Control supernatants were acquired from sterile medium. (**A**) Caspase activity assays were conducted on 4T1 cells after application of experimental supernatants or recombinant mouse TNF-*α* at 50 ng ml^−1^ for 24 h. Significant increases in caspase-3 and caspase-8 activities were observed after treatments with TNF-*α* and VNP pRA-TR supernatants when compared with controls (^*^P<0.05). Addition of the caspase-3 inhibitor, DEVD-fmk, significantly reduced activity. (**B**, **C**) Flow cytometry for annexin-V-FITC and propidium iodide was conducted on 10 000 4T1 cells per treatment in triplicate after application of experimental supernatants or TNF-*α* at 50 ng ml^−1^ for 48 h. (**B**) Flow cytometry dot plots after treatment with control (left) and VNP pRA-TR supernatants (right) indicate cell death (annexin-V and propidium iodide positive) proportions of 5.7 and 12.6%, respectively. (**C**) Results of flow cytometry indicate cell fractions undergoing cell death (annexin-V positive, propidium iodide positive) and early apoptosis (annexin-V positive, propidium iodide negative). Significant increases in cell death and early apoptosis were observed in 4T1 cells after treatment with the VNP pRA-TR supernatant (^*^P<0.05).

Supernatant fractions from the bacterial vectors for TRAIL delivery induced apoptosis and promoted cell death in 4T1 murine mammary carcinoma cells (Figure 2). Supernatants derived from VNP pRA-TR activated both caspase-8 and caspase-3, with responses similar to those observed for TNF-*α*, (*P*<0.05; Figure 2A). Addition of the caspase-3 inhibitor DEVD-fmk significantly reduced the activity of both caspases, confirming that the positive control, TNF-*α*, did activate caspase-3. Supernatants from the negative control, VNP pRA-ZsG, did not activate any of the two caspases (Figure 2A). Because the only difference between VNP pRA-TR and pRA-ZsG was the mTRAIL gene, the different cellular responses to the bacterial supernatants indicate that activation of the death receptor pathway was specifically dependent on mTRAIL expression. Annexin-V/propidium iodide flow cytometry showed that bacterial supernatants from VNP pRA-TR promote 4T1 cell death (annexin-V positive, propidium iodide positive) and early apoptosis (annexin-V positive, propidium iodide negative; Figures 2B and C). The proportion of cells undergoing cell death was increased in comparison with the control group (*P*<0.05), with more robust results than TNF-*α*. In addition, VNP pRA-TR supernatants significantly increased the annexin-V-positive fraction of cells (*P*<0.05; Figure 2C), indicating the early apoptotic stages of phosphatidylserine membrane translocation (Corsten *et al*, 2006).

Different modes of action confer a functional benefit of TRAIL over TNF-*α*. Although both act through the death receptor pathway, TNF-*α* stimulates both pro-apoptotic signals (through caspase-8) and mitochondrial-stabilising anti-apoptotic signals (through NF-κB), leading to cells with resistance to TNF-*α*-mediated apoptosis (Beg and Baltimore, 1996). TRAIL has attenuated the induction of NF-κB, leading to a pronounced death signal through p53-independent mechanisms (LeBlanc and Ashkenazi, 2003). This may explain the increase in early apoptosis, but overall lack of change in 4T1 cell death observed with TNF-*α* in Figure 2C.

### *S. typhimurium* VNP20009 preferentially colonises tumours

**Figure 3:**
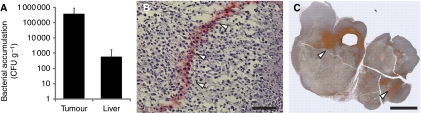
Spatial distribution of *S. typhimurium* after systemic administration. (**A**) Bacterial concentration (cfu g^−1^) in 4T1 mammary tumours and livers of BALB/c mice at 48 h after systemic injection of 100 000 cfu g^−1^ VNP20009. (**B**) Band of bacteria in a 4T1 tumour (stained red with white arrows) identified by anti-*Salmonella* immunohistochemistry. Scale bar is 100 *μ*m. (**C**) Composite image of 4T1 tumour stained using Salmonella immunohistochemistry to visualise bacteria (red with white arrows). Scale bar is 5 mm.

In a syngeneic subcutaneous mammary carcinoma model, systemic inoculation of 100 000 cfu g^−1^ VNP20009 resulted in the preferential colonisation of bacteria within 4T1 mammary tumours as compared with liver (Figure 3A). The average tumour density was almost 1000-fold greater than the liver density, which is similar to the numbers obtained in previous studies (Forbes *et al*, 2003). At both macro- and microscopic length scales, *Salmonella* were present in all tumours that were analysed (Figures 3B and C). At 48 h after administration, VNP20009 preferentially accumulated in necrotic regions of 4T1 tumours (white arrows, Figure 3C).

### *S. typhimurium* expressing TRAIL retard tumour growth in mice

**Figure 4:**
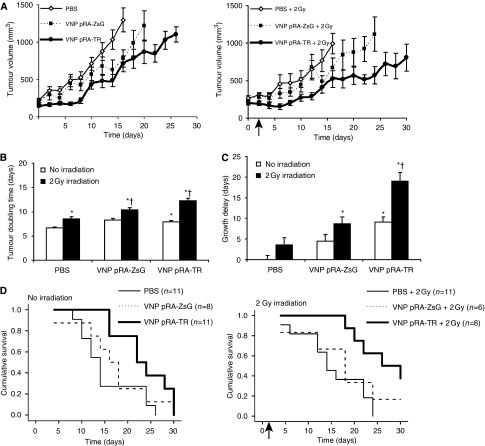
Reduced tumour growth and enhanced survival from VNP pRA-TR with induction by *γ*-irradiation. Balb/c mice received intravenous injection of 100 000 cfu g^−1^ VNP pRA-ZsG, VNP pRA-TR, or PBS at 21 days after establishing 4T1/red tumours. (**A**) Tumour volumes (mm^3^) after experimental treatments in mice receiving no irradiation, and in mice receiving 2 Gy irradiation on day 2 (arrow). No significant differences were observed in initial tumour volume between treatment groups. Mean tumour volume did not exceed 1000 mm^3^ in mice treated with VNP pRA-TR and 2 Gy at 1 month. (**B**) Tumour doubling time and (**C**) growth delay, as determined from regression analysis of exponential tumour growth curves. Growth delay was determined from the calculated time to tumour volume of 1000 mm^3^, normalised against the PBS control. ^*^P<0.05 compared with PBS. ^†^*P*<0.05 compared with PBS and 2 Gy. (**D**) Kaplan–Meier survival curves after experimental treatments in mice receiving no irradiation, and in mice receiving 2 Gy irradiation on day 2 (arrow). Survival analysis was based on follow-up until death or killing. Significant differences in 30-day survival were observed between mice receiving VNP pRA-TR and PBS, and between mice receiving VNP pRA-TR and 2 Gy and PBS and 2 Gy (log-rank test, *P*<0.05).

Treatment with bacterial vectors and *γ*-irradiation synergistically delayed tumour growth in 4T1/red tumour-bearing BALB/c mice (Figure 4; Table 1). Mice were systemically injected with PBS controls, control bacteria (VNP pRA-ZsG), or TRAIL-expressing bacteria (VNP pRA-TR), both with and without activation by 2 Gy *γ*-irradiation at 48 h after infection. All treatments significantly increased tumour doubling time compared with PBS controls (*P*<0.05; Figure 4B; Table 1). At all time points, except the day of injection, the tumour volumes of VNP pRA-TR with 2 Gy radiation were significantly less than PBS controls (*P*<0.05; Figure 4A). The combination of VNP pRA-TR with 2 Gy radiation increased the tumour doubling time from 6.6 days (95% CI 6.4–6.9 days) to 12.2 days (95% CI 11.7–12.8 days; *P*<0.05; Figure 4B) and caused a significant delay of 19.0 days (95% CI 15.0–23.1 days) for tumour growth to 1000 mm^3^ compared with the PBS controls (*P*<0.05; Figure 4C).

The delay in tumour growth for VNP pRA-TR with 2 Gy radiation was significantly greater than the control treatments, VNP pRA-ZsG with 2 Gy irradiation and VNP pRA-TR alone (Figure 4C; *P*<0.05), indicating that both TRAIL expression and induction by radiation were necessary for maximum growth delay. The significant difference in growth delay between irradiated VNP pRA-TR and VNP pRA-ZsG (*P*<0.05; Figure 4C) indicates that TRAIL is produced in tumours. Although this evidence is indirect, the only difference between the plasmids on these two strains is the TRAIL gene. If TRAIL was not produced, these two strains would have induced the same response. Compared with VNP pRA-TR with 2 Gy irradiation, radiation alone only delayed tumour growth by 3.5 days (95% CI 0.1–7 days), whereas VNP pRA-TR alone delayed growth by 9.1 days (95% CI 6.7–11.7 days), suggesting that the combined response was synergistic rather than additive. This synergy shows that there is activation of TRAIL expression by the *RecA* promoter as well as stimulation of bystander effects by the combination of radiation and TRAIL.

In addition to retarding growth, treatment with VNP pRA-TR and radiation improved mouse survival (Figure 4D; Table 2). To maintain animals according to humane principles, death was not used as a primary end point. For survival analysis, mice were killed when moribund or when tumours measured >1000 mm^3^. Although there was no difference in median survival between PBS with and without irradiation, median survival was nearly doubled by VNP pRA-TR with radiation (26 days) compared with PBS controls (14 days), with a significant increase in the 30-day survival from 0% to 37.5% of mice (log-rank test, *P*<0.05). Treatment with VNP pRA-TR and 2 Gy radiation reduced the risk of death by 76% when controlled against radiation treatment alone (hazard ratio 0.24; 95% CI 0.08–0.75; *P*<0.05).

### Redosing bacteria and irradiation accentuated the therapeutic effect

**Figure 5:**
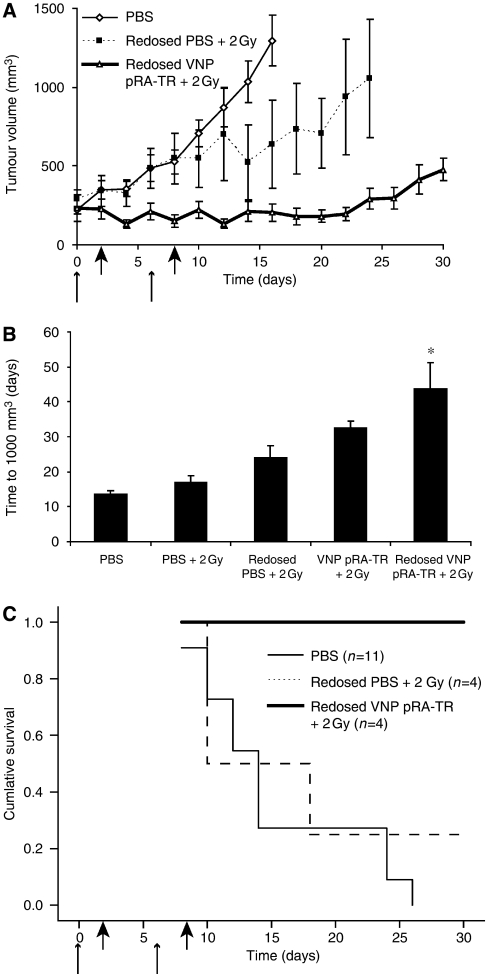
Suppression of tumour growth and enhanced survival by repeated dosing of VNP pRA-TR and *γ*-irradiation. (**A**) Regression curves for tumour growth after treatment with two doses of VNP pRA-TR and radiation (*n*=4), two doses of PBS and radiation (*n*=4), and PBS alone (*n*=11). Experimental groups received intravenous injection with PBS or VNP pRA-TR at days 0 and 6 (small arrows), followed by 2 Gy irradiation 2 days after experimental treatments (days 2 and 8; large arrows). Significant differences in tumour volume after redosing VNP pRA-TR and radiation were observed at all time points except days 0 and 2 in comparison with the PBS control group (*P*<0.05). (**B**) Estimates of time to tumour volume of 1000 mm^3^ show a delay of 30 days after two doses of VNP pRA-TR with irradiation in comparison with the PBS control (^*^P<0.05; redosed VNP pRA-TR+2 Gy, in comparison with all other groups). (**C**) Kaplan–Meier survival curves for the treatment groups in (A). At 30 days of follow-up, 100% of mice survived with two treatments of VNP pRA-TR with 2 Gy irradiation, compared with 25% after repeated dosing with PBS and 2 Gy, and no survival from PBS controls (log-rank test, *P*<0.05).

Because a single dose of VNP pRA-TR activated by 2 Gy radiation showed decreased growth for approximately 1 week, we reasoned that additional redosing at 1 week would potentiate the therapeutic effect (Figure 5). Additional mice received a series of two treatments, with intravenous injection of VNP pRA-TR or PBS on days 0 and 6, followed by 2 Gy radiation on days 2 and 8 (Figure 5A). The effects of redosing VNP pRA-TR with radiation were considerable, delaying the expected time to tumour volume of 1000 mm^3^ by 30.3 days (95% CI 26.6–34.1 days; *P*<0.05; Figure 5B; Table 3). The 30-day survival was 100% after redosing with VNP pRA-TR and radiation, compared with 25% after redosing with PBS and radiation (log-rank test, *P*<0.05), and 0% with PBS alone (*P*<0.05; Figure 5C).

## Discussion

These results show that *S. typhimurium* can express and secrete a functional TRAIL protein, with control of expression by genotoxic damage from a small dose of radiation. Secreted TRAIL, by activating the death receptor pathway, can stimulate apoptosis in carcinoma cells. Furthermore, *S. typhimurium* bearing pRA-TR significantly decreases tumour growth and increases survival when activated by radiation; an effect amplified by repeated dosing.

Complementary to these direct results, there are several added benefits in our approach. Numerous tactics have been developed with the intention of purposefully targeting cancers, including viral gene therapy, liposomal delivery systems, monoclonal antibodies, small-molecule protein inhibitors, and nanoparticle technologies; however, the ability to achieve spatial and temporal control while minimising toxicity has been limited. The first benefit of our approach is the ability of *S. typhimurium* to effectively target tumour microenvironments, with the ability to induce apoptosis within regions of quiescence bordering tumour necrosis (Kasinskas and Forbes, 2007). *S. typhimurium* has both tumour tropism and microenvironment specificity, with patterns of distribution within tumour microenvironments that are time dependent, offering opportunity for both spatial and temporal control of peptide delivery. The second benefit is the ability of TRAIL to target cancer cells with specificity, allowing for induction of apoptosis by p53-independent mechanisms while avoiding toxicity to the host observed with TNF-*α* (Takeda *et al*, 2007). The third benefit is the ability of the *RecA* promoter to act as a switch, turned on by genotoxic damage from radiation, allowing for temporal and spatial control of gene expression (Mitchell and Gu, 2004). Fourth, radiation therapy can potentiate responses to TRAIL delivery and influence the tumour microenvironment through bystander effects (Belka *et al*, 2004; Mothersill *et al*, 2004). Finally, repeated dosing in the setting of limited toxicity may provide a method to limit exponential growth in tumours. Once fully developed, we envision that this bacterial cancer therapeutic with spatial and temporal control of delivery will provide considerable therapeutic benefit by enhancing efficacy while limiting host toxicity.

## Figures and Tables

**Table 1:** Regression analysis for treatment effects on tumor growth

**Treatment**	***n***	**Growth curves, *V*(*t*)**	***r***	**Tumor doubling time (*d*)**	**Growth delay (*d*)**
PBS	11	ln(*V*)=0.10*t*+5.50	0.99	6.6 (6.4–6.9)	0 (0–2.0)
PBS+2 Gy	11	ln(*V*)=0.08*t*+5.52	0.98	8.5 (8.2–9.0)^*^	3.5 (0.1–7.0)
VNP pRZ	8	ln(*V*)=0.08*t*+5.40	0.98	8.3 (7.9–8.7)^*^	4.5 (1.3–7.6)
VNP pRZ+2 Gy	6	ln(*V*)=0.07*t*+5.44	0.98	10.4 (10.1–10.8)^*^^,†^	8.6 (5.3–12.0)^*^
VNP pRT	11	ln(*V*)=0.09*t*+4.92	0.97	7.9 (7.6–8.2)^*^	9.1 (6.7–11.6)^*^
VNP pRT+2 Gy	8	ln(*V*)=0.06*t*+5.07	0.95	12.2 (11.7–12.8)^*^^,†^	19.0 (15.0–23.1)^*^^,†^

Data are listed as means with their 95% confidence intervals in parentheses. Treatments of Balb/c mice with 21-day 4T1/red tumors were performed using intravenous injections of PBS (control), 100 000 cfu g^−1^ VNP20009 pRecA_ZsGreen (VNP pRZ), or 100 000 cfu g^−1^ VNP20009 pRecA_TRAIL (VNP pRT). Regression growth curves summarise volume (*V*, mm^3^) dependence on time (*t*, days) from initial treatment, with correlation coefficients indicated by *r*. Primary treatment dosing was at day 0, with or without 2 Gy irradiation at day 2. Tumor doubling time was derived from exponential growth curves. Growth delay was determined by assessing the time interval to 1000 mm^3^ compared with the PBS control.

^*^P<0.05 compared with PBS.

^†^*P*<0.05 compared with PBS and 2 Gy irradiation.

**Table 2:** Cox proportional hazards analysis for treatment effects on survival

**Treatment**	***n***	**Median survival (*d*)**	**Hazard ratios**	**Hazard ratios, stratified by radiation treatment**
PBS	11	14.0 (11.7–16.3)	—	—
PBS+2 Gy	11	14.0 (9.7–18.3)	0.97 (0.42–2.24)	—
VNP pRZ	8	16.0 (12.3–19.7)	0.63 (0.24–1.65)	0.58 (0.22–1.55)
VNP pRZ+2 Gy	6	18.0 (11.2–24.8)	0.53 (0.18–1.54)	0.58 (0.20–1.74)
VNP pRT	11	22.0 (14.6–29.4)	0.41 (0.16–1.04)	0.38 (0.15–1.00)
VNP pRT+2 Gy	8	26.0 (14.9–37.1)	0.21 (0.07–0.63)^*^	0.24 (0.08–0.75)^*^

Median survival times and hazard ratios are listed with their 95% confidence intervals in parentheses.

^*^Significance is indicated as *P*<0.05.

**Table 3:** Effects of treatment redosing

**Treatment**	***n***	**Growth curves, *V(t)***	***r***	**Tumor doubling time (*d*)**	**Growth delay (*d*)**	**30-day survival**
PBS	11	ln(*V*)=0.10*t*+5.50	0.99	6.6 (6.4–6.9)	0 (0–2.0)	0.0
PBS+2 Gy (2X)	4	ln(*V*)=0.05*t*+5.76	0.91	14.5 (13.7–15.3)^*^	10.5 (9.1–12.0)^*^	0.25
VNP pRT+2 Gy (2X)	4	*V*=0.07*t*^*2*^–16.67*t*+245.45	0.92	NA	30.3 (26.6–34.1)^*^^,†^	1.0^*^^,†^

Data are listed as means with their 95% confidence intervals in parentheses. Treatments of Balb/c mice with 21-day 4T1/red tumors were performed using intravenous injections of PBS (control) or 100 000 cfu g^−1^ VNP20009 pRecA_TRAIL (VNP pRT). Treatment dosing was at days 0 and 6, with 2 Gy irradiation at days 2 and 8. Regression growth curves summarise volume (*V*, mm^3^) dependence on time (*t*, days) from initial treatment, with correlation coefficients indicated by *r*. Tumor doubling time was derived from exponential growth curves. Growth delay was determined by assessing the time interval to 1000 mm^3^ compared with the PBS control. Note that for comparison, the PBS control data are repeated from Table 1.

^*^P<0.05 compared with PBS.

^†^*P*<0.05 compared with PBS and 2 Gy (2 × ) irradiation.
